# Shorter term aerobic exercise improves brain, cognition, and cardiovascular fitness in aging

**DOI:** 10.3389/fnagi.2013.00075

**Published:** 2013-11-12

**Authors:** Sandra B. Chapman, Sina Aslan, Jeffrey S. Spence, Laura F. DeFina, Molly W. Keebler, Nyaz Didehbani, Hanzhang Lu

**Affiliations:** ^1^Center for BrainHealth®, The University of Texas at DallasDallas, TX, USA; ^2^Advance MRI, LLCFrisco, TX, USA; ^3^The Cooper InstituteDallas, TX, USA; ^4^Advanced Imaging Research Center, The University of Texas Southwestern Medical CenterDallas, TX, USA

**Keywords:** aging, CBF, exercise, memory, MRI

## Abstract

Physical exercise, particularly aerobic exercise, is documented as providing a low cost regimen to counter well-documented cognitive declines including memory, executive function, visuospatial skills, and processing speed in normally aging adults. Prior aging studies focused largely on the effects of medium to long term (>6 months) exercise training; however, the shorter term effects have not been studied. In the present study, we examined changes in brain blood flow, cognition, and fitness in 37 cognitively healthy sedentary adults (57–75 years of age) who were randomized into physical training or a wait-list control group. The physical training group received supervised aerobic exercise for 3 sessions per week 1 h each for 12 weeks. Participants' cognitive, cardiovascular fitness and resting cerebral blood flow (CBF) were assessed at baseline (T1), mid (T2), and post-training (T3). We found higher resting CBF in the anterior cingulate region in the physical training group as compared to the control group from T1 to T3. Cognitive gains were manifested in the exercise group's improved immediate and delayed memory performance from T1 to T3 which also showed a significant positive association with increases in both left and right hippocampal CBF identified earlier in the time course at T2. Additionally, the two cardiovascular parameters, VO_2_ max and rating of perceived exertion (RPE) showed gains, compared to the control group. These data suggest that even shorter term aerobic exercise can facilitate neuroplasticity to reduce both the biological and cognitive consequences of aging to benefit brain health in sedentary adults.

## Introduction

The benefits of exercise for cardiovascular function are well established (Steinhaus et al., 1988; Ades et al., 2011). Research reveals that increased cardiovascular fitness can serve to reduce both the neurobiological and cognitive consequences of age-related declines (Colcombe et al., 2004; Macintosh et al., 2012). Physical exercise, particularly aerobic exercise, shows promise as a low-cost regimen to improve cognitive processes such as memory and executive functions in middle-age to older adults (Shay and Roth, 1992; Colcombe et al., 2006; Buchman et al., 2007; Erickson et al., 2011). Benefits of exercise are emerging in the neural mechanisms that support better learning and cognitive performance in aging. Aging studies have focused largely on the effects of extended (>6 months) exercise programs. Colcombe et al. (2004) found 6 months of aerobic exercise corresponded to improved cardiovascular fitness, better cognitive performance on executive function/attentional control, and increased task-related brain activity with reduced activity in the anterior cingulate. Another investigation showed a reversal of 1–2 years from the typical hippocampal volume loss associated with aging following 1 year of aerobic training as compared to a stretching-type exercise control group (Erickson et al., 2011). Moreover, a recent investigation identified elevated brain health markers in older master athletes (mean age of 74.5 ± 5.8 years) as manifested by a higher resting cerebral blood flow (CBF) in their posterior cingulate/precuneus regions (key nodes of default mode network) when compared to sedentary older adults (Thomas et al., 2013). In contrast to established gains for longer periods of exercise, little is known about the cognitive and brain plasticity gains possible in healthy but sedentary seniors from shorter term aerobic exercise training.

In the present study, we evaluated whether shorter term exercise (3 months) engenders gains in three domains: brain blood flow, cognition, and cardiovascular fitness. The study design was a randomized trial in which sedentary participants were randomly assigned to receive either aerobic training 3 sessions per week, 1 h per session for 12 weeks, or to be wait-listed controls. We investigated resting CBF, a dynamic physiological parameter and sensitive biomarker of cerebrovascular health and neuronal activity, via pseudo-continuous arterial spin labeling (pCASL) MRI (Aslan et al., 2010). Based on previous research showing that exercise increased cerebral blood volume (Pereira et al., 2007), perfusion of the hippocampus (Burdette et al., 2010) and improved memory (Erickson et al., 2011), we hypothesized that 12 weeks of physical training would increase CBF in hippocampus proper, improve cognition in the domains of memory/executive functions and enhance VO_2_max a physiological measure of cardiorespiratory fitness. Whereas extant research has shown physical exercise benefits the cardiovascular system, the relationships of enhanced physical fitness and improved cognitive function to changes in the cerebrovascular system are less well known (Shibata and Levine, 2012; Thomas et al., 2013). Our study further examined the relationships among physiological and cognitive gains with regional CBF measures. We hypothesized that aerobic exercise would promote brain health by direct links across cognition, cardiovascular function, and brain blood flow.

## Materials and methods

### Participants

A total of 37 cognitively normal adults (mean age = 64.0 ± 3.9, 57–75 years of age) were randomized into two different groups: physical training or wait-list control. Prior to randomization, all potential participants were carefully screened using the Telephone Interview Cognitive Status-Modified (TICS-M) to screen for dementia, the Montreal Cognitive Assessment (MoCA) to detect early cognitive impairment, the Beck Depression Inventory-II (BDI) to screen for depressive symptoms, and the Wechsler Abbreviated Scale of Intelligence (WASI) to assess estimated Intelligence Quotient (IQ). The participants also completed a thorough medical history, physical examination, anthropometric measurements, laboratory testing including fasting blood glucose/thyroid stimulating hormone (TSH), and an electrocardiogram. Finally, the participants underwent a maximal oxygen consumption (relative VO_2_ Max: $\frac{\text{mL}}{\text{kg}\times \text{ min}}$) exercise stress test to assess maximal exercise capacity as well as blood pressure/ECG responses and rating of perceived exertion (RPE) according to the Borg scale (range: 6–20) (Borg, 1990).

Inclusion criteria included: no prior history of neurological or psychiatric conditions, average IQ range, native English speaker, and minimum of a high school diploma. Exclusionary criteria included: MR scanning contraindications, cognitive impairment (TICS-M < 28 and MoCA < 26), elevated depressive symptoms (BDI >14), left-handedness, increased body mass $\left(\text{BMI}>40,\text{ BMI}=\frac{\text{mass}(\text{kg})}{\text{height}{\left(\text{m}\right)}^{2}}\right)$, abnormal electrocardiographic response, significant hypertensive blood pressure response to exercise, or inability to reach 85% of maximum predicted heart rate for age. Participants were also excluded if they reported regular aerobic activity of more than twice a week for 20 min or more. They could not have regularly exercised for at least 3 months prior to enrolling in the study. The sedentary lifestyle was confirmed independently by personnel at Cooper Institute and BrainHealth. Written informed consent was obtained from all subjects in accordance with the Institutional Review Board (IRB) of our academic institutions: The University of Texas at Dallas, The University of Texas Southwestern Medical Center, and The Cooper Institute.

### Physical training program

The physical training program was designed in an effort to ensure the participants met the 2008 physical activity guidelines of 150 min per week, as recommended for sedentary adults. The training regimen consisted of three 60 min sessions of aerobic exercise training per week for a period of 12 weeks. The participants' aerobic exercise alternated each session between exercise bike and treadmill. The exercise bike routine included: 5 min warm up at 43 watts, cycling for 50 min at a speed that increased their heart rate to 50–75% of their maximum achieved heart rate on VO_2_max testing, and a 5 min cool down at 43 watts. The treadmill workout included: 5 min warm up at 2 miles per h (mph), walking on treadmill for 50 min at a speed that increased their heart rate to 50–75% of their maximum achieved heart rate on VO_2_max testing, and a 5 min cool down at 2 mph. Every exercise session was supervised by an exercise physiologist and a nurse practitioner to ensure participants reached their target heart rate. A physician was also present onsite in case of emergencies.

### Physiological measures

Physiological markers were assessed and documented at The Cooper Institute at three time points: baseline/pre-training (T1), mid-training, week 6 (T2), and upon completion of the training, week 12 (T3). The physiological parameters included: weight (kg), heart rate (beats per min), VO2 Max (ml/kg/min), and RPE (Borg Scale: 6–20). The RPE was assessed based on Borg's Scale: 6 (no exertion) to 20 (extremely difficult).

### Neurocognitive measures

A battery of neurocognitive measures was administered at three time periods, i.e., baseline/pre-training (T1), mid-training, week 6 (T2), and upon completion of the training, week 12 (T3) for both control and training groups. Measurements of cognitive functions included tests of executive function, memory, and complex attention. Measures of executive function included: “Trails B-Trails A” (switching). Memory was assessed with trial one of the California Verbal Learning Test Second Edition (CVLT-II)—2 versions used alternately over time intervals, and immediate/delayed memory with Wechsler Memory Scale-Fourth Edition (WMS-IV). Complex attention was evaluated with Delis-Kaplan Executive Function System-Color Word Interference subtest (DKEFS-color word) and Backward Digit Span.

### MRI acquisition

MRI scans were performed on a 3 Tesla MR system (Philips Medical System, Best, Netherlands). A body coil was used for radiofrequency (RF) transmission and an 8-channel head coil with parallel imaging capability was used for signal reception. Foam padding was used to stabilize the head to minimize motion. A high resolution T1 weighted image was also acquired as an anatomical reference and a pCASL sequence (Aslan et al., 2010) was used to measure global/local CBF at baseline (T1), mid-training (T2), and at the end of training (T3). Imaging parameters for pCASL experiments were: single-shot gradient-echo EPI, field-of-view (FOV) = 240 × 240, matrix = 80 × 80, voxel size = 3 × 3 mm^2^, 27 slices acquired in ascending order, slice thickness = 5 mm, no gap between slices, labeling duration = 1650 ms, post labeling delay = 1525 ms, TR/TE = 4020/14 ms, SENSE factor 2.5, time interval between consecutive slice acquisitions = 35.5 ms, number of controls/labels = 30 pairs, RF duration = 0.5 ms, pause between RF pulses = 0.5 ms, labeling pulse flip angle = 18°, bandwidth = 2.7 kHz, echo train length = 35, and scan duration 4.5 min. The high resolution T1 weighted image parameters were: Magnetization Prepared Rapid Acquisition of Gradient Echo (MPRAGE) sequence, TR/TE = 8.3/3.8 ms, shot interval = 2100 ms, inversion time = 1100 ms, flip angle = 12°, 160 sagittal slices, voxel size = 1 × 1 × 1 mm^3^, FOV = 256 × 256 × 160 mm^3^, and duration 4 min.

### MR data processing

PCASL image series were realigned to the first volume for motion correction (SPM5's realign function, University College London, UK). Participants with head motion of >3 mm and >3° were excluded from further analysis. An in-house MATLAB (Mathworks, Natick, MA) program was used to calculate the difference between averaged control and label images. Then, the difference image was corrected for imaging slice delay time to yield CBF-weight image, which was normalized to the Brain template from Montreal Neurological Institute (MNI). This procedure was carried out using a non-linear elastic registration algorithm, Hierarchial Attribute Matching Mechanism for Elastic Registration (HAMMER, University of Pennsylvania, PA). The HAMMER algorithm detects and corrects for region-specific brain atrophy which is commonly seen in elderly subjects. Last, the absolute CBF (aCBF) was estimated by using Alsop and Detre's equation in the units of mL blood/min/100 g of brain tissue (Alsop and Detre, 1996). This method is represented by the following equation:

$$\begin{array}{l} f_{pCASL}(x,y,z)=\frac{\lambda \cdot e^{\left(\frac{\delta }{T_{1a}}\right)}}{-2\alpha \cdot M_{b}^{0}\cdot T_{1}\cdot \left[e^{\left(\frac{\min(\delta \;-\;w_{z}),0}{T_{1}}\right)}-e^{\left(\frac{-w_{z}}{T_{1}}\right)\left(1-\frac{T_{1RF}}{T_{1}}\right)}\right]}\\ \;\times \Delta M(x,y,z) \end{array}$$

where *f*_*pCASL*_ is the blood flow value at voxel (*x, y, z*) obtained from pCASL in ml blood/min/100 g brain, α is the labeling efficiency (0.86), λ is the blood-brain partition coefficient (0.98 ml/gram), δ is the arterial transit time of blood from the tagging plane to the imaging slice (2 s), *w* is the delay between the end of labeling and the start of acquisition (1.525 s), *T*_1_ is the brain tissue T_1_ (1.165 s), *T*_1*a*_ is the *T*_1_ of arterial blood (1.624 s), T_1*RF*_ is the T_1_ in the presence of off-resonance irradiation (0.75 s) and *M*^0^_*b*_ is the value of equilibrium magnetization of brain tissue, which was obtained from a manual region of interest (ROI) drawing of mid axial slice of the control image.

### Statistical analysis

The absolute whole brain blood flow values were calculated by averaging all the voxels in the aCBF map. The voxel wise and ROI analyses were performed on the relative CBF (rCBF) maps which included dividing the aCBF spatial maps by the whole brain aCBF value. In a prior publication, we had shown that such technique improves the sensitivity of regional differences by reducing physiological variations (Aslan and Lu, 2010). In the ROI analysis, the hippocampal and parahippocampal regions were defined and combined by a parcellation template in SPM (Tzourio-Mazoyer et al., 2002). In voxel based analyses (VBA), the individual rCBF maps were spatially smoothed [with full-width half-maximum (FWHM) of 4 mm] to account for small differences in sulci/gyri location across subjects. To define a threshold for the VBA results, we used a program based on AlphaSim (http://afni.nimh.nih.gov/pub/dist/doc/manual/AlphaSim.pdf), called *3dClustsim* in AFNI (NIMH Scientific and Statistical Computing Core, Bethesda, MD, USA), which controlled false positive activation clusters over the set of all activation clusters throughout the whole brain volume given voxel size, whole brain volume, and effective smoothness (inherent smoothness plus additional smoothness applied). We refer to this procedure in the Results as family-wise error correction (FWE corrected). The error correction was conditional on three criteria: smoothness of the voxel map, a magnitude threshold for defining clusters of contiguous voxels, and a minimum volume for each cluster. For the CBF VBA analysis, we used a FWE corrected *p* < 0.05, which is based on an effective smoothness of 8.8 mm FWHM, a cluster-defining magnitude corresponding to the 99.5th percentile of *t*-statistic distribution and a minimum volume of 83 voxels (664 mm^3^).

A general statistical linear model (GLM) was applied to assess the effects of physical training on CBF, neurocognitive, and physiological measures. For each of these dependent measures, the model included sessions (T1, T2, and T3), group status (physical training and control), as well as the interaction between these factors. We included two variance components in the model: one due to variability across subjects, and one due to variability in the same subject over time (repeated measures). We were primarily interested in how the groups differed across the training sessions (i.e., the interaction term in the model); thus, we hypothesized that the physical training group would show an improvement in mean measures, either by T2 or T3, relative to the control group. This hypothesis led to two orthogonal polynomial interaction contrasts: linear and quadratic. The *linear* interaction contrast tested whether the mean change between the groups increased monotonically from T1 to T3, and the *quadratic* interaction contrast tested whether the mean change between groups increased maximally at T2. All contrasts were scaled to t-statistics (implemented in SAS, Cary NC), and *p*-values were calculated with reference to Student's t-distribution on 69–70 degrees of freedom.

Significant clusters from the VBA, as well as a priori regions of interest, left/right hippocampus, were further examined for potential relationships with neuropsychological (NP) measures, cardiovascular measures (VO_2_ max), and RPE. These relationships were assessed in a GLM framework, similarly as above, with group status a classification variable, linear/quadratic contrasts of NP/VO_2_ max/RPE as a quantitative explanatory variable and linear/quadratic contrasts of rCBF estimates as dependent variables. Our primary interest was to determine whether these relationships between NP/VO_2_ max/RPE and rCBF differed between the physical training group and the controls (i.e., the interaction term, which allows separate relationships by group), and we hypothesized that the physical training group would demonstrate associations if they existed. As in the previous GLM, the regression coefficients as well as group differences between them were scaled to t-statistics with inference, similarly, based on Student's t-distribution on 30–32 degrees of freedom. All linear and quadratic estimates of these sets of variables were pair-wise tested; therefore, we applied the false discovery rate procedure (Benjamini and Hochberg, 1995) to minimize aberrant false positives which may plague the implementation of multiple tests. For similar reasons, we applied Bonferroni corrections by cognitive domain to the set of neurocognitive tests.

## Results

### Participant characteristics

All 19 control participants and 18 physical training participants completed the neuropsychological/physiological assessments at each time point. All physical training participants were required to complete at least 90% of physical training sessions over the 3 months training period. No participant was excluded due to missing too many sessions to meet the 90% criterion. One control participant did not complete all MRI protocol time points and was excluded from MRI analysis. No participant's imaging data had to be excluded based on motion. As a result, the final MRI data analyses were conducted on 18 control and 18 physical training participants for CBF. Table 1 summarizes demographic information, physical assessments, clinical evaluations, and total number of participants for the pCASL MRI protocol. No significant differences in age, gender, estimated IQ, MoCA, Telephone Interview of Cognitive Status-Modified (TICS-M) were noted between groups (*p* > 0.05).

**Table 1:** **Subject baseline characteristics and total number of subjects per assessment (mean ± *SD*)**.

	**Control**	**Physical training**
Age	64.0 ± 3.6	64.0 ± 4.3
Gender (M/F)	5/14	5/13
Estimated IQ	120.5 ± 10.6	117.5 ± 9.9
MoCA	28.2 ± 1.4	27.8 ± 1.5
TICS-M	29.6 ± 2.0	30.7 ± 2.0
Participants in physical training assessments	19	18
Participants in neuropsychological exams	19	18
Participants in pCASL MRI	18	18

IQ, Intelligence Quotient; MoCA, Montreal Cognitive Assessment; TICS-M, Telephone Interview of Cognitive Status-Modified.

### Physical training measures

As delineated in Table 2, several physiological parameters were measured at baseline, mid-, and end- of physical training to assess participants' fitness level. The VO_2_ max improved maximally at T2 in the physical training group relative to controls (*p* = 0.02), and the RPE improved from T1 to T3 in the physical training group compared to control group (*p* = 0.01). Table 2 summarizes the change in physiological parameters from baseline (T1) to mid (T2) and end point (T3) assessments.

**Table 2:** **Physiological measurements (mean ± s.e.m.)**.

**Physiological measures**	**Control**			**Physical training**			*p*-value^*^	
	**T1**	**T2**	**T3**	**T1**	**T2**	**T3**	**Linear**	**Quad**
Body weight (kg)	75.8 ± 3.2	75.7 ± 3.2	75.5 ± 3.2	77.2 ± 3.2	77.7 ± 3.2	77.5 ± 3.2	0.15	0.23
Max heart rate (bpm)	155.9 ± 3.1	154.0 ± 3.2	153.9 ± 3.4	152.4 ± 3.1	152.6 ± 3.2	152.6 ± 3.4	0.28	0.36
VO2 Max (ml/kg/min)	19.9 ± 0.9	19.7 ± 1.0	20.3 ± 1.0	19.3 ± 0.9	20.9 ± 1.0	20.4 ± 1.0	0.18	0.02
RPE	16.8 ± 0.4	17.2 ± 0.5	17.4 ± 0.4	16.2 ± 0.4	14.9 ± 0.5	15.3 ± 0.4	0.01	0.13

VO_2_ Max, Maximal Oxygen Consumption; RPE, Rating of Perceived Exertion.

* p-value refers to specified tests of interaction contrasts.

### Neurocognitive measures

Table 3 shows the neuropsychological exam results per the three cognitive domains for control and physical training groups. No significant differences were noted in the baseline scores (i.e., T1) between the physical training and control groups (*p* > 0.05). Tests of cognitive performance found that the physical training group significantly improved over training sessions in immediate and delayed text-level memory relative to the control group. Specifically, two measures of memory function, i.e., immediate and delayed text memory recall, showed improvement from T1 to T3 (*p* = 0.003 and *p* = 0.03, respectively).

**Table 3:** **Neuropsychological exam results (mean ± s.e.m.)**.

**Cognitive domain**	**Control**			**Physical training**			*p*-value^*^	
	**T1**	**T2**	**T3**	**T1**	**T2**	**T3**	**Linear**	**Quad**
**EXECUTIVE FUNCTION**								
“Trails B–Trails A” (rs)	28.5 ± 3.6	27.3 ± 3.7	31.2 ± 3.3	29.6 ± 3.4	28.4 ± 3.8	30.0 ± 3.4	0.61	0.85
**MEMORY**								
CVLT-II trial 1 (rs)	7.5 ± 0.4	6.9 ± 0.4	7.7 ± 0.6	6.9 ± 0.5	6.7 ± 0.5	7.7 ± 0.5	0.50	0.91
LM immediate (rs)	17.1 ± 0.7	14.5 ± 0.7	14.8 ± 0.8	15.2 ± 0.7	15.7 ± 0.7	16.8 ± 0.8	0.003	0.24
LM delayed (rs)	15.6 ± 0.7	14.3 ± 0.9	15.3 ± 0.8	13.5 ± 0.7	14.4 ± 0.9	15.5 ± 0.8	0.03	0.32
**COMPLEX ATTENTION**								
Stroop condition 3 (rs)	55.3 ± 2.8	51.6 ± 2.1	50.7 ± 2.1	61.1 ± 2.9	56.8 ± 2.2	56.5 ± 2.6	0.98	0.72
Stroop condition 4 (rs)	61.5 ± 3.0	56.8 ± 2.2	56.5 ± 2.6	61.5 ± 3.0	58.9 ± 2.3	56.1 ± 2.4	0.69	0.76
Backward digit span (ss)	8.0 ± 0.5	8.5 ± 0.5	8.7 ± 0.6	7.2 ± 0.5	7.6 ± 0.5	7.5 ± 0.6	0.53	0.90

California Verbal Learning Test-II, LM, Logical Memory; ss, scaled score; rs, raw score.

* p-value refers to specified tests of interaction contrasts.

**Figure 1:**
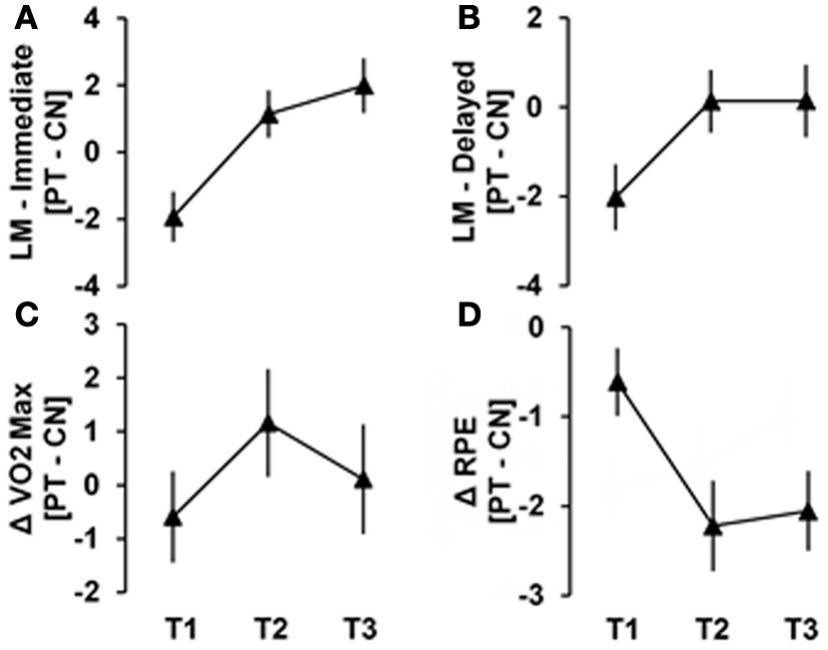
**The mean difference between Physical Training (PT) and Control (CN) groups over training sessions are shown for (A) immediate logical memory (B) delayed logical memory (C) VO_2_ Max and (D) rating of perceived exertion (RPE)**. Significant changes from T1 to T3 are evident in **(A, B, D)** (*p* = 0.003, 0.03, and 0.01, respectively). Maximal change at T2 is evident for panel **(C)** (*p* = 0.02).

Figure 1 shows the mean change plot between physical training (PT) and control (CN) groups over training sessions. The physical training group showed significant increases relative to the control group from T1 to T3 in immediate logical memory (*p* = 0.003) and delayed logical memory (*p* = 0.03), a significant decrease relative to the control group from T1 to T3 in RPE (*p* = 0.01) and a significant maximal increase at T2 relative to the control group in VO2 max (*p* = 0.02). In each of these cases, with the exception of immediate logical memory, the physical training group alone contributed to the significant interactions: VO_2_ max (*p* = 0.005 for PT, *p* = 0.447 for CN), RPE (*p* = 0.035 for PT, *p* = 0.274 for CN), and LM delayed (*p* = 0.003 for PT, *p* = 0.750 for CN). Finally, for LM immediate, the physical trainers alone had a significant increase from T1 to T3 (*p* = 0.013), while the controls had a significant decrease from T1 to T3 (*p* = 0.007).

### MRI measurements

aCBF was measured by pCASL MRI in both control and physical training groups at rest. The global CBF at T1 for both control and physical training groups were similar (47.1 ml/100 g/min and 46.8 ml/100 g/min, respectively, *p* = 0.91) and did not change significantly across time points (Linear: *p* = 0.39, Quad: *p* = 0.19). To evaluate local resting CBF changes, we conducted ROI and voxel-wise analyses on rCBF. The rCBF of the hippocampus did not change significantly between control (rCBF of T1/T2/T3: 1.18/1.18/1.20) and physical training groups (rCBF of T1/T2/T3: 1.17/1.19/1.21), Linear: *p* = 0.42 and Quad: *p* = 0.45.

**Figure 2:**
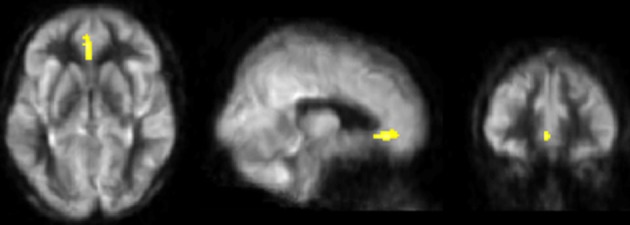
**Results of CBF voxel based comparison superimposed on an average CBF map of all participants**. Anterior cingulate cortex's CBF increased from T1 to T3 in the physical training group (shown in yellow) compared to the control group, *p* < 0.05 (FWE corrected) and *k* ≥ 664 mm^3^.

Figure 2 shows the whole-brain results from the VBA. The physical training group showed an increase in resting-state blood flow from T1 to T3 in bilateral anterior cingulate (ACC) compared to the control group (FWE corrected *p* < 0.05). Table 4 summarizes ACC finding.

**Table 4:** **CBF regions that showed significant blood flow change at rest in physical training and control groups from T1 to T3**.

**Brain regions**	**BA**	**Cluster size (mm^3^)**	***MNI***			***T*-value**
			***X***	***Y***	***Z***	
**LINEAR: EXERCISE > CONTROL**						
L/R anterior cingulate	32	696	0	34	−8	3.90

### Neural correlates of brain, cognition, and cardiovascular changes

**Figure 3:**
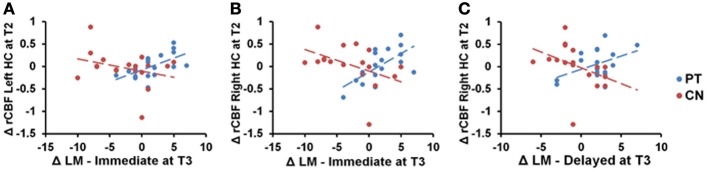
**Scatterplots of immediate logical memory (LM) change from T1 to T3 against maximal T2 change of rCBF in the hippocampus (HC)**. The physical training group shows positive relationships between LM immediate scores and bilateral hippocampus that differ significantly from controls [Panel **(A)**: *p* = 0.015; Panel **(B)**: *p* < 0.001]. Panel **(C)** shows a similar relationship between delayed LM scores and right hippocampus (*p* = 0.022).

Figure 3 shows the scatterplots of relationships between logical memory changes and hippocampal CBF changes. The increase in immediate logical memory scores from T1 to T3 is strongly associated with a maximal T2 change in hippocampal rCBF. A positive relationship was evident in physical trainers in both the left and right hippocampus (*p* = 0.025 and *p* < 0.001, respectively), while the relationships for controls were slightly negative (*p* = 0.129 and *p* = 0.023, respectively). Moreover, the stated relationships for the physical trainers were significantly more positive than for controls in both the left and right hippocampus (*p* = 0.015 and *p* < 0.001, respectively). Similarly, the positive relationship between delayed logical memory changes and right hippocampus rCBF changes for the physical trainers was significantly more positive than the relationship for the controls (*p* = 0.070 for PT, *p* = 0.060 for CN, *p* = 0.022 for the difference). Last, we found a positive trend for the physical trainers between anterior cingulate cortex from T1 to T3 and VO_2_ Max at maximal T2 (*p* = 0.13) as well as a negative trend for the physical trainers between anterior cingulate cortex from T1 to T3 and RPE from T1 to T3 (*p* = 0.11). However, neither of these hypothesized results was significant following false discovery rate control.

As suggested by an anonymous reviewer, we additionally ran the above tests in a separate brain region than the one hypothesized to be impacted by exercise, left/right caudate, without specific hypotheses of neuropsychological and physical relationships with blood flow, to garner support for hippocampal specificity. In the left caudate we found no significant linear relationships with logical memory scores in either group (LM immediate—*p* = 0.235 for CN and *p* = 0.104 for PT; LM delayed—*p* = 0.277 for CN and *p* = 0.315 for PT). Similarly, we found no significant linear relationships in the right caudate (LM immediate—*p* = 0.661 for CN and *p* = 0.131 for PT; LM delayed—*p* = 0.621 for CN and *p* = 0.964 for PT).

## Discussion

Brain and cognitive functions decline as early as the third decade in otherwise healthy adults (Schaie and Strother, 1968; Salthouse, 2009; Chen et al., 2011; Lu et al., 2011). The present research suggested that aerobic exercise in sedentary adults can improve cognitive/brain health. Physical activity augmented three domains; brain function (resting regional CBF), cognition (i.e., immediate/delayed memory), and cardiovascular fitness (VO_2_max and RPE). The benefits were measured after shorter term exercise (i.e., 3 months), extending prior evidence of longer-term exercise gains (>3 months) (Colcombe et al., 2004, 2006; Hillman et al., 2008; Burdette et al., 2010; Voss et al., 2010), including benefits from being life-long exercisers as manifested in master-athletes (Thomas et al., 2013). The participants showed gains as early as 6 weeks with continued gains at 12 weeks. A relatively rapid health benefit across brain, memory, and fitness in sedentary adults soon after starting to exercise, some gains starting as early as 6 weeks, could motivate adults to start exercising regularly (Kramer et al., 2005; Pereira et al., 2007; Smith et al., 2010).

The current findings shed new light on ways exercise promotes cognitive/brain health in aging. These data provide some of the first evidence integrating significant associations across exercise-induced improvements in cerebrovascular and cardiovascular measures, along with CBF increases in ACC, as well as improved fitness levels and gains in verbal memory linked to elevated CBF in hippocampi. The resulting patterns can be interpreted by considering several factors: (1) regional nature of neural networks, (2) time course of changes (3) differences in brain measures and training duration. The brain regions selectively enhanced by exercise are still being unveiled. Whereas enhancement of hippocampal regions has been a consistent prior finding (Burdette et al., 2010; Voss et al., 2010; Erickson et al., 2011), a novel finding was the increased rCBF in the ACC. The regional brain changes are consistent with prior evidence that exercise is associated with a selective rather than global impact on brain (Burdette et al., 2010; Prakash et al., 2010).

Our findings of a positive correlation between increased CBF in left/right hippocampi at T2 and improved immediate/delayed memory at T3 suggest the brain-behavior relationship is a meaningful gain. We propose that exercise training first increased the CBF and then lead to memory performance improvement. Additionally, our findings of significant rCBF changes in the ACC may precede changes to hippocampal structural changes, identified in longer exercise programs (Erickson et al., 2011). The changes to ACC and hippocampal regions may be linked since these regions purportedly represent closely connected neighborhood network nodes that may be beneficially impacted with exercise (Burdette et al., 2010). Burdette and colleagues found a major increase in connectivity between hippocampi and the ACC in an exercise trained group. Our data would support their findings that the ACC and hippocampi could become increasingly well-connected to each other following exercise-training. Favorable changes to these regions following exercise is of interest due to accruing data these regions are detrimentally impacted by aging and dementia (Gusnard et al., 2001; Greicius et al., 2004). The hippocampus CBF did not show an increase in our study compared to Burdette et al.'s which may be due to several reasons. First, the average age of Burdette's exercise group was 14 years older than our group (78 vs. 64 years old). It may be that physical exercise has different effects on different age groups. Second, the participants in our study were sedentary whereas the participants in Burdette's study were excluded only if they actively participated in a formal exercise program (>30 min/week) 1 month prior to study recruitment. Third, the duration of Burdette's exercise training was 1 month longer compared to ours (4 vs. 3 months). Last, Burdette used pulsed arterial spin labeling (PASL) MRI technique with 60 label/control pairs whereas we used pCASL MRI technique with 30 label/control pairs. Additionally, the CBF maps quantification was not identical.

Another contribution is data supporting the potential for CBF in ACC and hippocampal regions to be sensitive markers in older adults of gains in cognitive brain health. Prior researchers have suggested that reduced hippocampal blood flow could provide informative measures to track declines in performance with aging (Heo et al., 2010). Our data support CBF as a potential index of gains, not just declines. Moreover, evidence that exercise could have a direct benefit to CBF in the ACC is pertinent to improved cognitive brain health. Harrison et al. (2012) found prominence of the ACC as measured by cortical brain volume in unusually successful cognitive agers (>80 years) compared to middle-aged/ elderly controls who show the predicted decline (Harrison et al., 2012). Additionally, the ACC has been linked to strategic gating of information for memory (Mesulam et al., 2001). For example, the ACC has been associated with (1) better monitoring of memory (Risius et al., 2013), (2) allocation of attention supporting memory (Kondo et al., 2004), and a critical node in working memory (Kondo et al., 2004; Engstrom et al., 2013).

We interpret our findings of seemingly disparate findings from Colcombe et al. (2004) who reported a reverse pattern of ACC activity using fMRI activation trials. Their results revealed less activity in the ACC accompanied by increased activity in the frontal and parietal/precuneus with exercise training. Explainable differences observed between task-based fMRI studies (Colcombe et al., 2004) vs. resting-state studies within a functional neural circuit is supported by the work of Biswal et al. (2010). Biswal and colleagues showed a dynamic differential change within individuals in neural activity in the same brain regions between vigilant-rest vs. active-task performance. Based on this evidence, one plausible explanation for the separate patterns may be that higher resting CBF in the ACC would, in the context of task-related activity, afford task-related skill-development and, hence, more efficient brain functions during actual task performance. Future efforts are needed to illuminate the resting vs. activation of ACC in adults who are high-capacity memory performers vs. those showing pathological brain changes of disease.

Elevated regional CBF may be an earlier index of positive effects of exercise than measures of brain structure, thus accounting for CBF changes after only 6 weeks of physical training. Changes in CBF reportedly emerge earlier than structural brain changes in response to either training gains or disease-driven losses (Bruel-Jungerman et al., 2007; Smith et al., 2010). Pereira et al. (2007) found increased cerebral blood volume after 3 months of exercise training similar to our finding. Erickson et al. reported structural changes after 1 year of exercise training in sedentary adults. Thus, patterns of regional brain changes may depend on duration of exercise training as well as the brain measurement employed.

We propose that sedentary adults experienced positive brain, cognitive, and physical gains after 3 months of aerobic training for several reasons. First, our data show a significant correspondence between regional hippocampus CBF changes and memory as well as increased CBF in ACC. While we found increases in both cerebral and peripheral vasculature health, the correspondence between the two (i.e., higher CBF in ACC and VO_2_ max) failed to reach significance. The correspondence between these two domains continues to be of interest. For example, do peripheral gains from exercise in cardiovascular functionality serve to alter intrinsic brain systems through CBF or do brain changes exert a top-down control influence over cardiovascular systems. We interpret our findings cautiously but suggest that the influence of exercise training impacts the cardiovascular system more globally with primarily selective impact on the cerebrovascular system. Similar to Thomas et al. (2013), we did not find a difference in whole-brain CBF, but rather regional changes in CBF. Elevating whole-brain CBF in adults may require increased neural activity through cognitive challenges, whereby through neurovascular coupling, the cerebrovasculature is impacted more globally with complex cognitive training than can be achieved by physical training alone (Chapman et al., 2013). Future studies are needed to investigate the differential impact of exercise vs. cognitive training. The peripheral vascular system shows dramatic changes during exercise whereas the cerebral cortex exhibits only modest changes in CBF compared to resting state (Thomas et al., 2013).

The present findings are novel, however we acknowledge that the resulting patterns across brain, cognition, and physical fitness domains are primarily correlational and thus do not elucidate a clear mechanism of how exercise brings about improvement in these three domains. Nonetheless, extant evidence indicates that exercise training serves to improve many factors associated with improved health including anti-inflammatory benefits (Kadoglou et al., 2012), protective effects on the vascular system (Conti and Macchi, 2013), improvement in slow wave sleep (Horne, 2013), as well as enhanced mood (Blumenthal et al., 1999). One clear trend is that, as a society, we are becoming increasingly more sedentary compared to earlier in our evolutionary history, when physical activity was inherent in everyday life habits. Sedentary habits are taking its toll on our cardiovascular and brain health (Conti and Macchi, 2013). From the present study, benefits are realized after 3 months of training with the possibility of other beneficial effects that were not measured.

This study has several limitations. One is the small sample size, another is lack of active control group, and a third is lack of follow-up. The cardiorespiratory fitness improvement at T2 was sustained at T3, albeit at a slightly lower level that is not clinically meaningful. While this may be related to small sample size, there is also a known variability in individual response to exercise training (Bouchard and Rankinen, 2001). Future work should evaluate the dose response relationship between exercise, cardiorespiratory fitness, and brain variables. The lack of an active control group in the present study does not allow us to rule out the possibility that some benefits may arise from purely social engagement rather than strictly to the physical training alone. Future trials should include an active control group which could either include a non-aerobic type of exercise such as yoga or a class where individuals interact with some form of non-exercise coaching. Because our results align with prior findings of exercise benefiting memory, we feel the results are likely to be upheld in the context of an active control group. We note that despite the small sample size and lack of active control group, the significant and convergent findings across brain imaging, cognitive, and cardiovascular gains support the claim that the physical training program has the potential to significantly improve brain, cognitive, and physiological performance in sedentary but otherwise healthy middle age to young seniors.

## Conclusions

This study adds new insights into the mounting evidence of benefits from aerobic training revealing benefits across cardiovascular fitness, cognition, and regional CBF in adults. The significant gains in the anterior cingulate region are intriguing since this region has recently been linked to superior cognitive-agers in late life. Heretofore, most studies showed the gains after long term training typically were achieved after 6 months or more. The present study showed gains across three domains earlier than previously documented in sedentary middle-aged to old adults. The findings suggest that healthy life style changes in exercise habits can help to mitigate unnecessary losses. The sooner one starts the better since the slope of declines in brain and cognitive health become steeper from age 50 forward (Dregan and Gulliford, 2013).

## Author contributions

Sandra B. Chapman designed the study, interpreted the data and drafted the manuscript, Sina Aslan performed neuroimaging analysis, interpretation of neuroimaging data and drafted the manuscript; Jeffrey S. Spence performed statistical analysis and critical review of the manuscript; Laura F. DeFina supervised physical training sessions and critical review of the manuscript; Molly W. Keebler and Nyaz Didehbani performed cognitive assessments and reviewed the manuscript; Hanzhang Lu designed the neuroimaging protocols and critical review of the manuscript.

### Conflict of interest statement

The authors declare that the research was conducted in the absence of any commercial or financial relationships that could be construed as a potential conflict of interest.
